# The USDA cucumber (*Cucumis sativus* L.) collection: genetic diversity, population structure, genome-wide association studies, and core collection development

**DOI:** 10.1038/s41438-018-0080-8

**Published:** 2018-10-01

**Authors:** Xin Wang, Kan Bao, Umesh K. Reddy, Yang Bai, Sue A. Hammar, Chen Jiao, Todd C. Wehner, Axel O. Ramírez-Madera, Yiqun Weng, Rebecca Grumet, Zhangjun Fei

**Affiliations:** 1 Boyce Thompson Institute, Cornell University, Ithaca, NY 14853 USA; 2 Gus R. Douglass Institute and Department of Biology, West Virginia State University, Institute, Virginia, WV 25112 USA; 3 Department of Horticulture, Michigan State University, East Lansing, MI 48824 USA; 4 Horticultural Science Department, North Carolina State University, Raleigh, NC 27695 USA; 5 Horticulture Department, University of Wisconsin, Madison, WI 53706 USA; 6 USDA-ARS Vegetable Crops Research Unit, Madison, WI 53706 USA; 7 USDA-ARS Robert W. Holley Center for Agriculture and Health, Ithaca, NY 14853 USA

## Abstract

Germplasm collections are a crucial resource to conserve natural genetic diversity and provide a source of novel traits essential for sustained crop improvement. Optimal collection, preservation and utilization of these materials depends upon knowledge of the genetic variation present within the collection. Here we use the high-throughput genotyping-by-sequencing (GBS) technology to characterize the United States National Plant Germplasm System (NPGS) collection of cucumber (*Cucumis sativus* L.). The GBS data, derived from 1234 cucumber accessions, provided more than 23 K high-quality single-nucleotide polymorphisms (SNPs) that are well distributed at high density in the genome (~1 SNP/10.6 kb). The SNP markers were used to characterize genetic diversity, population structure, phylogenetic relationships, linkage disequilibrium, and population differentiation of the NPGS cucumber collection. These results, providing detailed genetic analysis of the U.S. cucumber collection, complement NPGS descriptive information regarding geographic origin and phenotypic characterization. We also identified genome regions significantly associated with 13 horticulturally important traits through genome-wide association studies (GWAS). Finally, we developed a molecularly informed, publicly accessible core collection of 395 accessions that represents at least 96% of the genetic variation present in the NPGS. Collectively, the information obtained from the GBS data enabled deep insight into the diversity present and genetic relationships among accessions within the collection, and will provide a valuable resource for genetic analyses, gene discovery, crop improvement, and germplasm preservation.

## Cucumber: Core collection for future crop improvement

Genetic analysis of the US cucumber collection provides valuable insights into the plant’s diversity and generates a core resource for future research. Breeding crop plants requires in-depth understanding of plant genetics and the identification of key genes that enhance traits such as disease and stress resistance. Zhangjun Fei at the Boyce Thompson Institute in Ithaca, Rebecca Grumet at Michigan State University, and co-workers across the US used high-throughput genetic sequencing to characterize the 1,234 cucumber accessions held by the US National Plant Germplasm System. The team uncovered 23,000 specific genome variations that revealed the genetic diversity, population structure and genetic differentiation of the cucumber. They identified genome regions associated with 13 traits important to cultivation and selected 395 accessions covering 96 percent of the collection’s genetic variation to make a publicly accessible core of data.

## Introduction

Improvements in crop yield, ability to withstand abiotic and biotic stresses, and superior product quality all depend on genetic variation for key agronomic and horticultural traits. In search of such variation, breeders frequently turn to germplasm collections to find new sources of valuable characteristics, especially resistances to diseases, insects, and environmental stresses such as heat, drought, salt, or cold. To facilitate these breeding efforts and maintain critical diversity for future generations, many national and international institutions have developed extensive germplasm collections to provide repositories of genetic variation. More than 1750 gene banks have been established worldwide^1^. Collections are typically made from locations throughout the globe, with particular emphasis on centers of crop diversity. The importance of such collections as a critical first step to conserve biological variation, especially in light of genetic erosion resulting from habitat loss, adoption of modern varieties, and climate change, is increasingly recognized as a critical global good, both in scientific and broader public spheres^2,3^. While creation and maintenance of these valuable collections is essential, questions arise as to how to catalog, unlock, manage, and preserve the valuable diversity they contain. How do we evaluate the extent and nature of variation that exists within the collection? How can we access that variation for crop improvement? Fortunately, the past decade has ushered in powerful genomic tools that allow for high throughput, high resolution, genetic characterization, while also providing breeders more efficient access to, and use of, the diversity available within collections.

Collections for the Cucurbitaceae family, which includes many high-value crops consumed as vegetables and fruits throughout the world, face the above-mentioned challenges for germplasm preservation and utilization^4^. Cucumber (*Cucumis sativus* L.), a member of the Cucurbitaceae family with origins in India, China, Burma, Thailand, is thought to have been domesticated ~3000 years ago^5,6^. The primary and secondary centers of diversity for the species are located in India and Southeast Asia, respectively^7,8^. Genomic analysis of cultivated cucumber (*C. s*. var. *sativus)* divided it into four geographic groups: India; Eurasia and the West; East Asia and China; and Xishuangbanna from Southwestern China^9,10^. The Indian group, which is thought to form the basal group, maintains a large proportion of the genetic diversity and also includes the wild cucumber, *C. s*. var. *hardwickii*, a feral form of var. *sativus*^9–11^. Deep resequencing of a core collection of 115 cucumber lines, sampled from 3342 accessions worldwide, suggests that the domestication process led to a severe genetic bottleneck, resulting in reduction in diversity relative to wild accessions^10^. More than 100 putative selective sweeps appear to be associated with domestication, including extended linkage disequilibrium in regions surrounding loci associated with key fruit traits such as size and bitterness. Results of the genomic analyses, including assignment of a basal role of the Indian group and separation of the orange-endocarp Xishuangbanna group, complement prior genetic and morphological assessments^12–16^. These analyses have allowed for evolutionary insight into the relationships and domestication trajectories among cucumber accessions.

The cucumber collection in the United States is maintained at the Ames, Iowa facility of the USDA Agriculture Research Service National Plant Germplasm System (NPGS; https://npgsweb.ars-grin.gov/gringlobal/site.aspx?id=16). The NPGS collection comprises 1314 cucumber accessions representing the primary cucumber gene pool (*C. s*. var. *sativus* and *C. s*. var. *hardwickii*). This collection, which is primarily composed of cultivars, land races, and varieties collected from around the world, has been extensively utilized by breeders searching for a variety of traits, including resistance to downy mildew^17^ (causal agent: *Pseudoperonospora cubensis*), powdery mildew^18^ (causal agent: *Podosphaera xanthii*), *Phytophthora* fruit rot^19,20^ (causal agent: *Phytophthora capsici*), belly rot^21,22^ (causal agent: *Rhizoctonia solani*), and root knot nematodes (*Meloidogyne* spp.)^23^, as well as variations for fruit yield, fruit quality^24^, and above-ground and below-ground plant architecture^25,26^. However, to date, there have been very limited efforts to genetically characterize the US cucumber collection. Meglic et al.^27^ examined 757 accessions using seven isozyme loci, and Horejsi et al.^28^ characterized 118 accessions with 71 RAPD loci. Lv et al.^9^ included 883 accessions from the U.S. collection, which were characterized using a set of 23 SSR markers and 316 alleles. Current genomic technologies allow for much higher throughput and full genome analyses. The dramatically reduced cost of sequencing, high-throughput sample preparation, and efficient bioinformatics now make it feasible to perform genomic analysis on increasingly large numbers of samples for plant germplasm research^29,30^. In this study, we have performed genotyping on 1234 cucumber accessions from the NPGS, using genotyping-by-sequencing^30^ (GBS). The resultant high-throughput single-nucleotide polymorphism (SNP) markers provided high-definition genetic characterization of the US cucumber germplasm collection, allowing for assessment of genetic diversity and population structure, identification of markers that are highly associated with important agronomic traits through genome-wide association studies (GWAS), and development of a molecularly informed publicly accessible core population to facilitate breeding and preservation efforts.

## Materials and methods

### Plant materials and DNA extraction

Tissue samples (50–100 mg fresh weight) were collected from young (not fully expanded) leaves, freeze-dried, and ground to a fine powder using 5/32” stainless steel balls (AbbottBall, West Hartford, CT) in a Retsch Mixer Mill (Retsch, Newtown, PA). DNA was isolated using the Omega Mag-Bind Plant DNA DS Kit (M1130, Omega Bio-Tek, Norcross, GA) on a Kingfisher Flex Magnetic Particle Processor (Thermo Scientific, Waltham, MA). The kit protocol was followed except that the initial 56 °C incubation was extended to 60 min instead of 30 min. The DNA was quantified using the Quant-iT PicoGreen dsDNA Kit (Invitrogen, Carlsbad, CA) in a 384-well format on a CFX384 C1000 Real-Time thermal cycler (BioRad, Hercules, CA). Normalization to 30–100 ng/ul was done using a GBFit Arise Pipetting System (Pacgen Inc., Irvine, CA). Quality checks were performed on 10% of the genomic DNA samples from each batch of 96 samples by agarose gel observation of 300 ng of undigested and *Hind*III digested DNA per sample.

### GBS and SNP calling

Genotyping of the cucumber accessions was performed following the GBS protocol^30^, using *ApeK*I as the restriction enzyme. The resulting 96-plex or 384-plex libraries were sequenced on a HiSeq 2500 system (Illumina Inc., USA) with the single-end mode and read length of 101 bp.

SNP identification was performed using TASSEL 5.0 GBS Discovery Pipeline^31^, using the cucumber Gy14 draft genome (v2; http://cucurbitgenomics.org) as the reference. Briefly, the raw reads were first processed to retain reads possessing a barcode and a restriction enzyme cut site using GBSSeqToTagDBPlugin with the parameters “-kmerLength 90-minKemrL 30-mnQS 10-c 100-maKmerNum 200000000”. The resulting reads were then concatenated into distinct tags using the FastqToTagCount plug-in in TASSEL, and tags supported by at least ten reads were kept and mapped to the cucumber reference genome sequence using BWA (version 0.7.16a) with default parameters^32^. Based on the alignments, positions of aligned tags were determined using SAMtoGBSdbPlugin, and SNPs were identified from the aligned tags using DiscoverySNPCallerPluginV2 with default parameters. The identified SNPs were scored according to the coverage, depth, and genotypic statistics for a given set of samples using SNPQualityProfilerPlugin. SNPs were filtered based on their missing data rate and minor allele frequencies (MAF) using VCFtools^33^.

### Phylogenetic and population genomic analyses

SNPs with MAF ≥ 1% and missing data rate ≤ 50% were used for phylogenetic and population structure analyses. The maximum-likelihood (ML) phylogenetic tree was constructed using SNPhylo^34^ with parameters “-r -M 0.5 -m 0.01 -l 0.1 -B 100” and visualized using the ggtree package^35^. PI 618817 (*Cucumis myriocarpus*) and PI 282446 (*C. heptadactylus*) were used as the outgroup. Principal component analysis (PCA) was performed using Plink-1.9 (ref. ^36^). Population structure analysis was performed using the STRUCTURE program^37^. A total of 11,745 SNPs with linkage disequilibrium (LD) decay (*r*^*2*^) < 0.4 were used for the analysis. To determine the most likely group number, STRUCTURE was run 20 times using 8000 SNPs randomly selected from the 11,745 SNPs, for each K (*K* = 2–20). The highest *∆K*, which indicates the most likely number of clusters in the population, was obtained. After determining the best K (*K* = 3), we then ran STRUCTURE using all 11,745 SNPs with 10,000 iterations for each K (*K* = 2–4).

LD decay was measured by correlation coefficients (*r*^*2*^) for all pairs of SNPs within 500 kb that were calculated using PopLDdecay v3.27 (https://github.com/BGI-shenzhen/PopLDdecay) with the following parameters: -MaxDist 500 -MAF 0.05 -Het 0.88 -Miss 0.999. The maximum value of *r*^*2*^ was calculated using all pairs of SNPs within 500 bp. The nucleotide diversity (π) and population fixation index (*F*_ST_) were calculated using Bio:PopGen implemented in the BioPerl package^38^. To visualize the pairwise *F*_ST_ values among different groups, multidimensional scaling (MDS) was conducted using the cmdscale function in R to transform *F*_ST_ values into two-dimensional values, which were used for plotting.

### GWAS

The USDA-GRIN database archives phenotypic data for *Cucumis* (https://npgsweb.ars-grin.gov/gringlobal/cropdetail.aspx?type=descriptor&id=123). The phenotypic data of 13 important traits for cucumber, including three related to disease resistance (anthracnose, downy mildew, and gummy stem blight (GSB) resistance), three related to root knot nematode resistance (resistance to *Meloidogyne hapla* race 1, *M. arenaria* race 2, or *M. incognita* race 3), three related to fruit shelf life (weight loss, firmness loss, and shriveling), and four other traits (chilling tolerance, days to flower, root size, and fruit yield), were downloaded from the GRIN database. The phenotypic data were collected over the last 30 years by the Cucurbit Breeding program of North Carolina State University. Data sets for each trait were collected over multiple years and locations (http://cucurbitbreeding.com) for 750–950 cultigens per trait. Description of the data collection is available at Supplementary Note. Phenotypic data from accessions genotyped in the present study were used for GWAS.

We used a total of 72,982 biallelic SNPs without any filtering to construct the kinship (K) matrix, which was used to correct for population structure and kinship in the GWAS analyses. For GWAS, the missing genotypes in the raw biallelic SNP dataset were imputed using the k-nearest neighbor (KNN) algorithm implemented in the fillGenotype software^39^. In order to obtain the optimal imputation accuracy and filling rate, three accessions with few missing genotypes (Amex 7735, NSL 32744, and PI 167052) were selected and 10%, 20%, and 30% SNP sites were randomly masked as missing genotypes for imputing. The imputation was performed using the fillGenotype with the following parameters: w (20, 30, 50, 65, 80), p (−3, −5, −7, −9), k (3, 5, 7, 9), and *r* (0.65, 0.7, 0.75, 0.8). The optimal combination of parameters (w = 30, k = 9, *p* = −9, *r* = 0.8) was selected after comparing the filling rate and imputation accuracy of each combination of parameters, to impute the missing genotypes in the raw dataset. Only biallelic imputed SNPs with minor allele frequency ≥ 1% and missing data rate ≤ 20% (a total of 28,650 SNPs) were used for GWAS. GWAS were performed using the linear mixed model (LMM) implemented in Fast-LMM^40^. The genome-wide significance thresholds of the GWAS were determined using the Bonferroni correction at α = 0.05 for significant and *α* = 0.01 for extremely significant associations as described in Li et al.^41^. In this study, the significance thresholds of *α* = 0.05 and *α* = 0.01 corresponded to raw *P* values of 1.75 × 10^–6^ and 3.49 × 10^−7^, or −log10(*P*) values of 5.76 and 6.46, respectively.

### Core collection selection

GenoCore^42^ was used to select a subset of accessions that captured the majority of the allelic diversity of the 1234 cucumber accessions, with the following parameters: -d 0.01%, -cv 100%. Combined with phenotypic analysis, we obtained the final core collection containing 395 cucumber accessions, of which 354 were genotyped in the current study. The percentage of the allelic diversity captured by the 354 accessions in this core collection was determined using GenoCore. The core collection was further evaluated by PCA, using the same methods described above for the entire collection.

## Results

### Genotyping of cucumber germplasm collection and variation identification

**Fig. 1:**
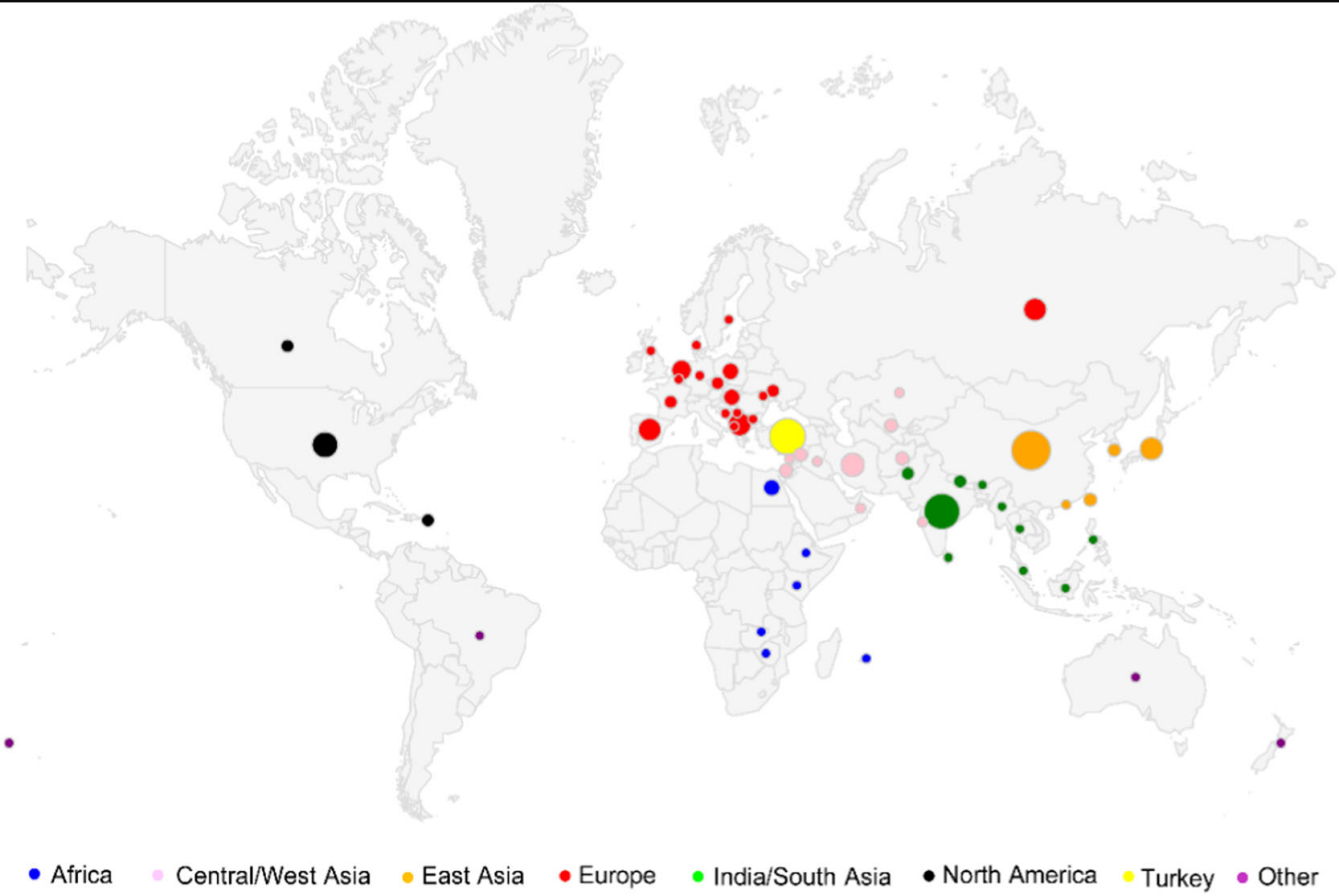
Geographical distribution of the 1234 cucumber accessions in the National Plant Germplasm System (NPGS). Size of the circles indicates the relative sampling size in each country

Seed was successfully germinated for 1234 cucumber accessions from the NPGS, which represents 94% of the collection (1314 accessions for which seed was available). Based on their geographic distribution (countries of origins), we classified these accessions mainly into seven groups, 216 from India/South Asia, 293 from East Asia, 113 from Central/West Asia, 161 from Turkey, 314 from Europe, 33 from Africa, 97 from North America, as well as 7 from other regions (Fig. 1 and Supplementary Table S1). The accessions from India (184) along with 32 plant introductions (PIs) from the surrounding regions of Bhutan, Malaysia, Nepal, Myanmar, Pakistan, Sri Lanka, and Thailand were classified separately from accessions from other Asian countries, since India and the surrounding regions are considered as the center of origin of cultivated cucumber^6,43^. The Indian/South Asia group also included three accessions of *C. s*. var. *hardwickii*. In addition, since Turkey is a country straddling Asia and Europe, we put accessions from Turkey as an independent group. We genotyped these cucumber accessions, as well as two non-cucumber but closely related accessions PI 618817 (*C. myriocarpus*) and PI 282446 (*C. heptadactylus*), using the GBS technology, which generated a total of ~1.35 billion reads of 101 bp in length. The numbers of reads for each sample ranged from ~176 K to 4.8 million, with a median of 677 K reads (Supplementary Table S1). A total of 554 K unique tags with at least 10 read counts, which corresponded to ~1.23 billion reads, were obtained and used for SNP calling. The 1.23 billion GBS reads were aligned to the reference Gy14 genome (version 2; http://cucurbitgenomics.org), with 55.5% (0.69 billion corresponding to 279 K tags) aligned to unique positions and 17.3% (0.21 billion corresponding to 50 K tags) to multiple locations; the remaining 27.2% (0.33 billion corresponding to 225 K tags) unaligned reads were mainly from mitochondrion and chloroplast, as well as genome regions that were absent in the reference genome. Approximately 3.7% (9.5 Mb out of 258.6 Mb) of the Gy14 genome was covered by the aligned GBS reads, which is typical for reduced complexity GBS data^30^.

**Fig. 2:**
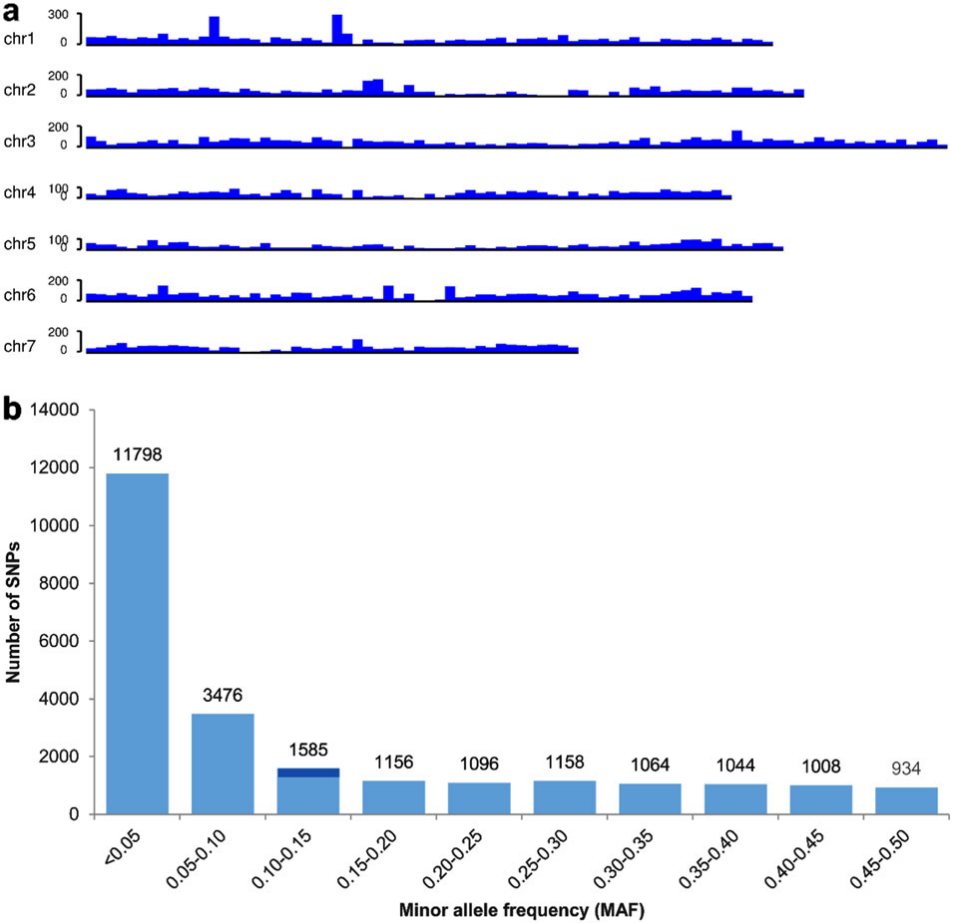
Cucumber SNPs. **a** SNP density across the seven cucumber chromosomes. Number of GBS-SNPs in each 500 kb non-overlapping window are shown. **b** Distribution of minor allele frequency (MAF) for the filtered SNPs

Based on the alignments, we identified a total of 114,760 variation sites, of which 113,854 were SNPs and 906 were small insertions/deletions (InDels). After retaining SNPs with ≤ 50% missing data (representing at least 617 accessions) and MAF ≥ 0.01 (i.e., SNPs present in at least 7 accessions), we obtained a total of 24,319 SNPs distributed across the cucumber Gy14 genome with an average of one SNP per 10.6 kb (Table 1). Only eight regions > 500 kb in the Gy14 genome were not covered by SNPs, and all these eight regions were centromeric or pericentromeric (Fig. 2a). The distribution of MAF of these SNPs is shown in Fig. 2b. The average MAF was 0.13; nearly half (11,798; 48.5%) of the SNPs had MAF between 0.01 and 0.05. Among the 24,319 SNPs with ≤ 50% missing data and MAF ≥ 0.01, only those that were biallelic were retained as the final SNP dataset (23,552 SNPs) used in the downstream analyses, unless otherwise specified.

**Table 1:** Summary statistics of the identified SNPs across each cucumber chromosome

**Chromosome**	**size (bp)**	**No. raw SNPs**	**Average raw SNP distance (bp)**	**No. filtered SNPs (<** **50% missing and MAF** **>** **0.01)**	**Average filtered SNP distance (bp)**
chr0	27,604,159	11,054	2497.2	2542	10,859.2
chr1	33,288,019	15,707	2119.3	3525	9443.4
chr2	35,008,273	13,954	2508.8	2953	11,855.2
chr3	41,698,299	19,794	2106.6	4195	9940.0
chr4	31,152,864	12,978	2400.4	2793	11,153.9
chr5	33,752,483	13,346	2529.0	2528	13,351.5
chr6	32,362,979	16,875	1917.8	3627	8922.8
chr7	23,759,298	10,146	2341.7	2156	11,020.1
Total	258,626,374	113,854	2271.6	24,319	10,634.7

### Phylogenetic relationships and population structure of the cucumber accessions

**Fig. 3:**
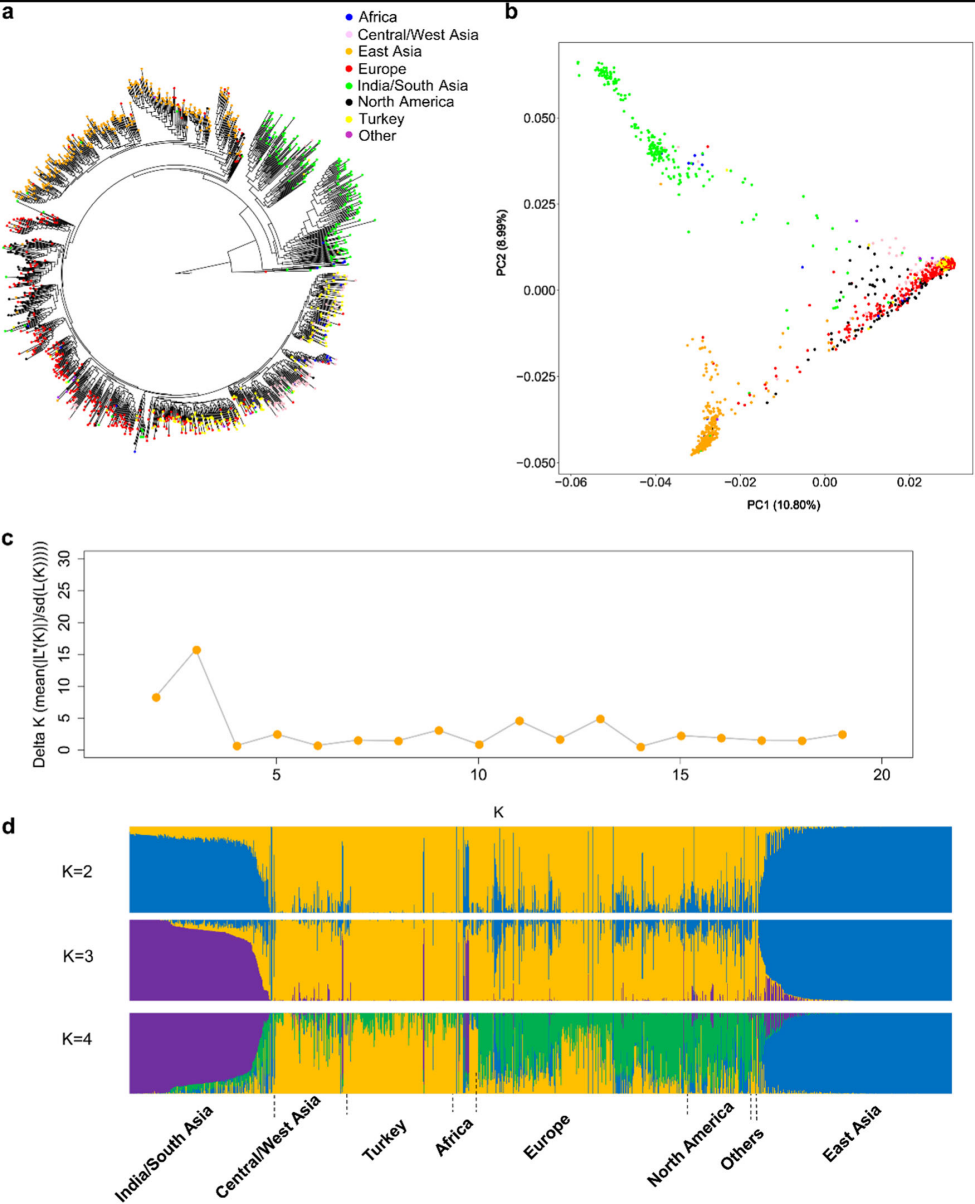
Phylogenetic and population genomic analyses of cucumber accessions. **a** Rooted maximum-likelihood phylogenetic tree of the 1234 cucumber accessions constructed using GBS-SNPs. PI 618817, *C. myriocarpus*, and PI 282446, *C. heptadactylus* were used as the outgroup. An enlarged version of the tree with searchable accession names is provided as Supplementary File 1 in pdf format. **b** Principal component analysis (PCA) of the 1234 cucumber accessions. The first two PCs explain about 20% of variance, with PC1 and PC2 explaining 10.80% and 8.99%, respectively. **c** Plot of *ΔK* values with *K* from 2 to 19 in the STRUCTURE analysis. **d** Population structure analysis of cucumber accessions with *K* from 2 and 4. Each accession is represented by a vertical bar. Each color represents one ancestral population, and the length of each colored segment in each vertical bar represents the proportion contributed by ancestral populations

Using the final SNP dataset, we constructed a rooted ML tree to infer phylogenetic relationships among the cucumber accessions, using PI 618817 (*C. myriocarpus*) and PI 282446 (*C. heptadactylus*) as the outgroup (Fig. 3a and Supplementary File 1). Three major clades were identified. Consistent with India as the center of origin for cucumber, the clade with the deepest branches was the India/South Asia group. The remaining accessions were separated into two major clades. One mainly contained accessions from East Asia, while the second encompassed accessions from Central/West Asia, Turkey, Europe, Africa, and North America.

PCA of these cucumber accessions illustrated a similar pattern of their phylogenetic relationships (Fig. 3b). Our results are consistent with those reported in Qi et al.^10^, which also classified cucumbers into three primary groups, with the exception of the Xishuangbanna group, for which no accessions were included in our GBS set.

To investigate the population structure of cucumber, the Bayesian clustering algorithm implemented in the STUCTURE program^37^ was first used to estimate ancestry proportions for each cucumber accession. ∆*K* analysis showed that three populations (*K* = 3) represented the best number of clusters for these 1234 cucumber accessions (Fig. 3c). As shown in Fig. 3d, at *K* = 2, accessions from East Asia and India were clearly separated from other accessions. At *K* = 3 (optimal), the India/South Asia group was clearly separated from the East Asia group. The population structure result at this optimal K was consistent with the phylogenetic tree and PCA results; all suggested three primary clusters in the cucumber accessions collected from NPGS. At *K* = 4, a new subpopulation emerged mainly in accessions from Europe and North America. A large portion of accessions from Europe, North America, Africa, Turkey, and Central/West Asia showed genetic admixture, while most of the East Asia accessions had a homogeneous genetic background.

Within the India/South Asia clade were several subclades (Fig. 3a and Supplementary Fig. S1). The Indian accessions within the U.S. NPGS were collected in two time periods: a first set of materials was entered into the system prior to 1972, and a second set collected in 1992. The accessions collected in 1992 were primarily from the states of Rajasthan, Uttar Pradesh, and Madhya Pradesh, representing regions in North and Central India that were largely missed in the prior collection^44^. The Indian accessions were differentially distributed among the different subclades, especially those from Rajasthan that were primarily associated with subclade 2, suggesting that the subclades, in part, reflect geographic distribution within India. Accessions from prior collections from South or Southwest India (Maharashtra, Karnataka, and Kerala) clustered in subclade 3. Subclade 1 primarily contained accessions from Madhya Pradesh in central India. The great majority of the East Asian accessions were collected from China. Those from Japan and South Korea largely clustered with each other; the remaining subclades were almost exclusively composed of accessions from China (Supplementary Fig. S1). For accessions from Turkey, two subclades were identified, one clustered with accessions from Central/West Asia group, and the other clustered with accessions from Europe (Supplementary Fig. S1). The North American accessions, also showed division into two distinct subclades. One group was largely comprised of pickling (processing) cultigens and the other of slicing (fresh market) cultigens (Supplementary Fig. S1), reflecting the two predominant market classes produced in the US.

### LD patterns, genetic diversity, and population differentiation in cucumber

The LD decay (*r*^*2*^) with increasing physical distance between SNPs was calculated for each group (Supplementary Fig. S2). When the entire population was analyzed, the average physical distance over which LD decayed to half of its maximum value was around 24 kb (*r*^*2*^ = 0.0930; maximum *r*^*2*^ = 0.1830). Variable LD decays were detected in different groups. The Africa group and the North American group had the longest physical distances over which LD decayed to half of its maximum value, 64 kb and 96 kb, respectively, while the India group had the shortest, 16 kb. The Europe, Turkey, East Asia, and the Central/West Asia groups showed comparable LD decay patterns and physical distances (48 kb, 40 kb, and 32 kb for Europe, East Asia, and Central/West Asia, respectively).

We then evaluated the genetic diversity within different groups. The average values of genome-wide nucleotide diversity (π) for Central/West Asia, Europe, North America, Africa, Turkey, East Asia, and India/South Asia groups were 0.87 × 10^−3^, 0.90 × 10^−3^, 0.93 × 10^−3^, 0.98 × 10^−3^, 0.81 × 10^−3^, 0.74 × 10^−3^, and 1.22 × 10^−3^, respectively. The π value of the India/South Asia group was higher than those of other groups, consistent with India being the center of origin of cultivated cucumber where cucumber accessions are expected to be more genetically diverse.

**Fig. 4:**
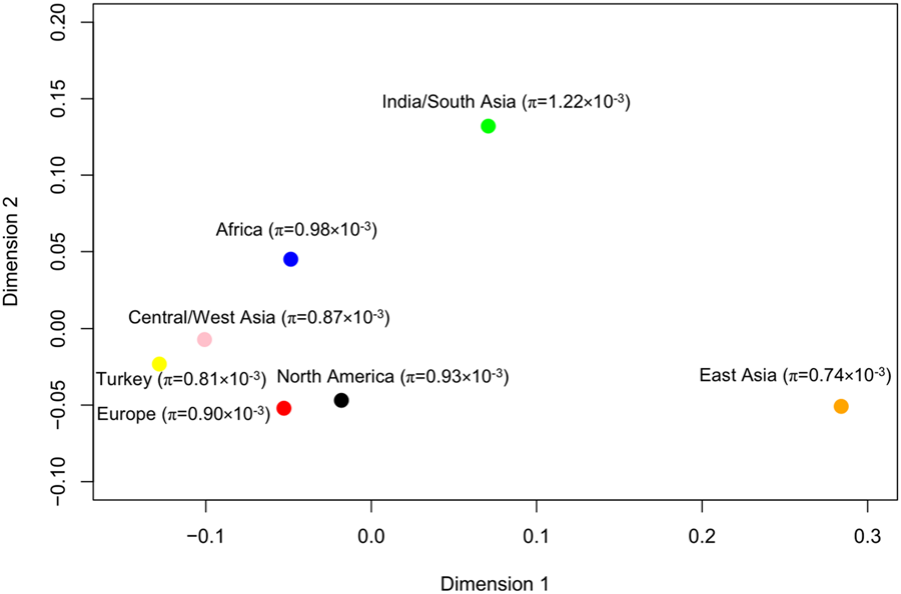
Multidimensional scaling of pairwise *F*_ST_ values between different cucumber groups

We further investigated population divergence among different groups by calculating pairwise fixation index (*F*_ST_) values. Pairwise weighted *F*_ST_ values among North America, Central/West Asia, Africa, Turkey, and Europe groups ranged from 0.042 to 0.14, while the values between East Asia and other six groups ranged from 0.284 to 0.413, and between India/South Asia and other six groups from 0.176 to 0.284 (Supplementary Table S2). Visualization of pairwise weighted *F*_ST_ values using MDS showed a clear distinction between the East Asia group and other groups. *F*_ST_ between East Asia vs. India/South Asia was 0.284 and between East Asia vs. the Western group (North America, Europe, Africa, and Central/West Asia) was 0.269. There was much less divergence among the North America, Europe, Turkey, Africa, and Central/West Asia groups, and between the Western group and India/South Asia (0.15) (Fig. 4). Collectively, both π and *F*_ST_ values suggested that domestication and improvement of cultivated cucumbers from Indian cucumbers occurred independently in East Asia compared to other regions.

### Genome-wide association studies in cucumber

The high-density SNP markers combined with GWAS provide a powerful resource to identify quantitative trait loci (QTL) and possible candidate genes for important horticultural traits. We collected historical phenotypic data of cucumber accessions from the NPGS for 13 agronomic traits, which included three traits related to disease resistance (anthracnose, downy mildew, and GSB resistance), three related to root knot nematode resistance (resistance to *Meloidogyne hapla* race 1, *M. arenaria* race 2, or *M. incognita* race 3), three related to fruit shelf life (weight loss, firmness loss, and shriveling), and four other traits (cold tolerance, days to flower, root size, and fruit yield). For each trait, data were available for around 600–700 accessions that were genotyped using GBS in this study (Supplementary Table S3). The phenotypic data largely followed normal distribution without significant skewness except for resistance to *M. hapla* race 1 (Supplementary Fig. S3). GWAS were performed for these traits with the imputed SNPs, which had an imputation accuracy of > 99% and missing data filling ratio of > 96.5% (Supplementary Table S4), using the LMM accounting for population structure and kinship. Significantly associated SNPs could be identified except for resistance to *M. incognita* race 3 and root size (Supplementary Table S5).

#### GWAS for disease and nematode resistance

**Fig. 5:**
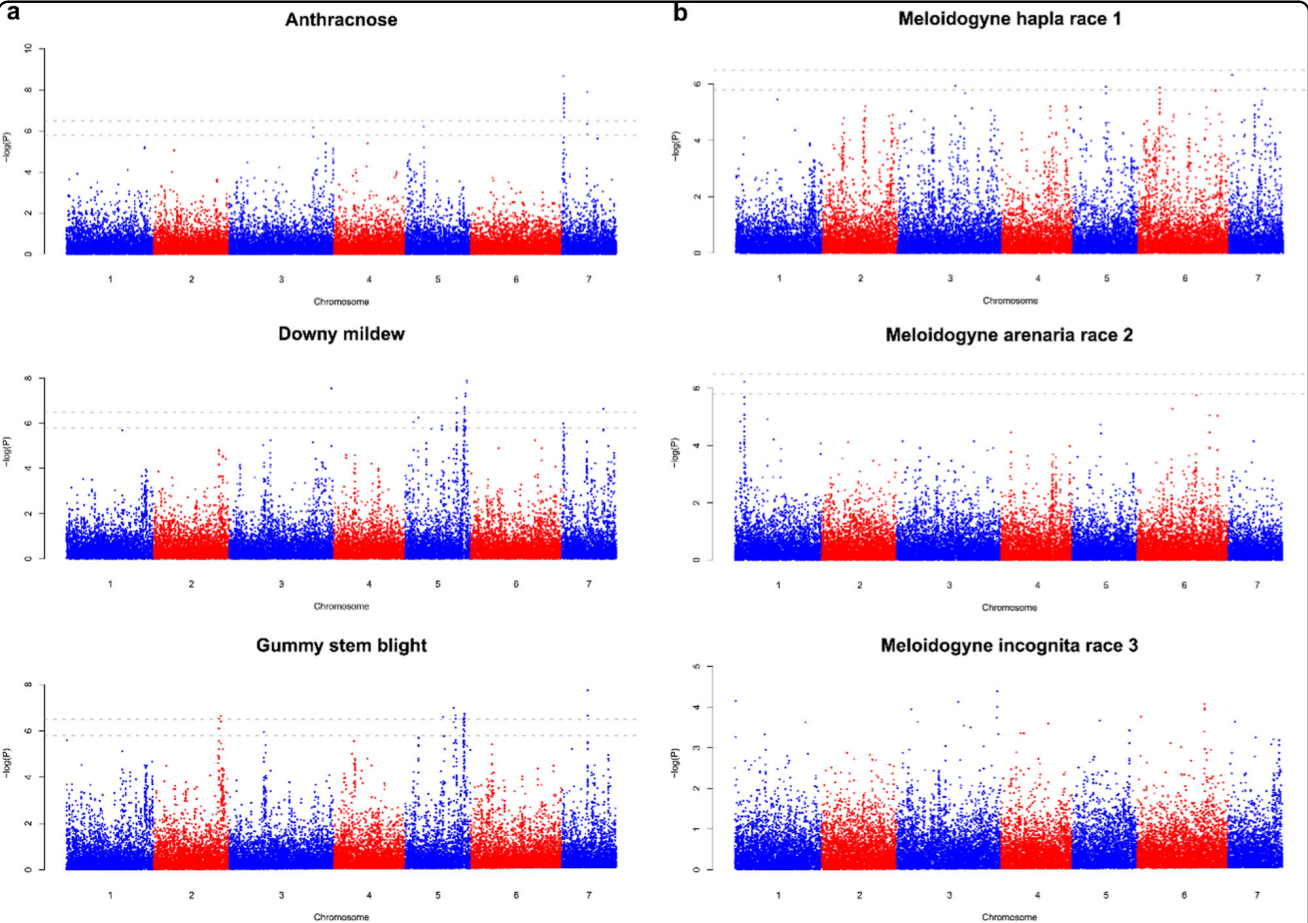
Manhattan plot of genome-wide association study (GWAS) results. **a** GWAS for disease resistance traits including resistance to downy mildew, anthracnose, or gummy stem blight. **b** GWAS for root knot nematode resistance traits including resistance to *Meloidogyne hapla* race 1, *M. arenaria* race 2, or *M. incognita* race 3

For anthracnose resistance, two regions on chromosome 7 were identified (Fig. 5a). A total of 11 SNPs spanning one region (from 1.0 to 1.1 Mb) and a total of five SNPs spanning another region (from 12.52 to 12.55 Mb) were found to be significantly associated with anthracnose resistance (Supplementary Table S5). Other significantly associated SNPs were identified at 33.1 Mb of chromosome 3 and 10.06 Mb of chromosome 5.

For downy mildew resistance, a region on chromosome 5 spanning from 29.38 to 32.46 Mb was identified to contain 27 significantly associated SNPs (Fig. 5a and Supplementary Table S5). Eight other SNPs significantly associated with downy mildew resistance were identified, with one on chromosome 3 (40.78 Mb), four on chromosome 5 (4.26, 6.54, 14.64, and 22.49 Mb), and three on chromosome 7 (9.55, 10.96, and 19.73 Mb).

For GSB resistance, three regions, one on chromosome 2, one on chromosome 5 and one on chromosome 7 were identified (Fig. 5a). The region on chromosome 2 spanned from 30.67 to 31.83 Mb and contained four significantly associated SNPs; the region on chromosome 5 spanned from 28.68 to 31.34 Mb and contained 25 significantly associated SNPs (Supplementary Table S5). Another two SNPs, on chromosome 3 (13.05 Mb) and 5 (23.05 Mb), respectively, were identified to be significantly associated with GSB resistance.

For root knot nematode resistance, no regions were identified to be significantly associated with resistance to *M. incognita* race 3; while a SNP on chromosome 1 (3.18 Mb) was identified to be significantly associated with resistance to *M. arenaria* race 2 (Fig. 5b). Six SNPs, one on chromosome 3 (26.48 Mb), one on chromosome 5 (19.67 Mb), two on chromosome 6 (7.37 and 28.88 Mb), and two on chromosome 7 (1.35 and 16.72 Mb) were significantly associated with resistance to *M. hapla* race 1 (Supplementary Table S5).

#### GWAS for fruit yield and physiological traits

Fruit yield trait in the cucumber accessions was investigated at two locations, Iowa and North Carolina. GWAS for fruit yield using data from each of the two locations as well as combined identified a total of nine significantly associated SNPs, one on chromosome 2 (30.07 Mb), two on chromosome 3 in a region at 27.86 Mb, three on chromosome 4 (27.00, 28.27 and 29.83 Mb), and three on chromosome 5 in a region spanning from 2.847 to 2.864 Mb (Supplementary Fig. S4a and Supplementary Table S5).

GWAS were performed for three traits related to fruit shelf life, weight loss, loss of firmness, and shriveling. Five SNPs, three on chromosome 4 (2.22 Mb and two at 28.78 Mb) and two on chromosome 7 (696 and 978 kb) were identified for weight loss, one SNP on chromosome 3 (27.23 Mb) was identified for loss of firmness, and one SNP on chromosome 2 (3.18 Mb) was identified for shriveling (Supplementary Fig. S4b and Supplementary Table S5).

For chilling tolerance, eighteen SNPs were identified, with ten on chromosome 1, one on chromosome 2, two on chromosome 4 and five on chromosome 7. For days to flower, six SNPs, one on chromosome 1, one on chromosome 3, two on chromosome 4, and two on chromosome 6 were identified (Supplementary Fig. S5 and Supplementary Table S5). No significant associations were found for root size.

### Development of a publicly accessible core cucumber germplasm collection

Our main objective of developing a core collection from the cucumber accessions in the NPGS is to provide the community with a subset of representative cucumber accessions that can be used for future GWAS, QTL mapping, marker development, and gene cloning studies. The selected core collection would have a reasonable size (~400 accessions) and capture largely the allelic diversity of the entire collection, and also include accessions with some unique and important agronomic traits.

**Fig. 6:**
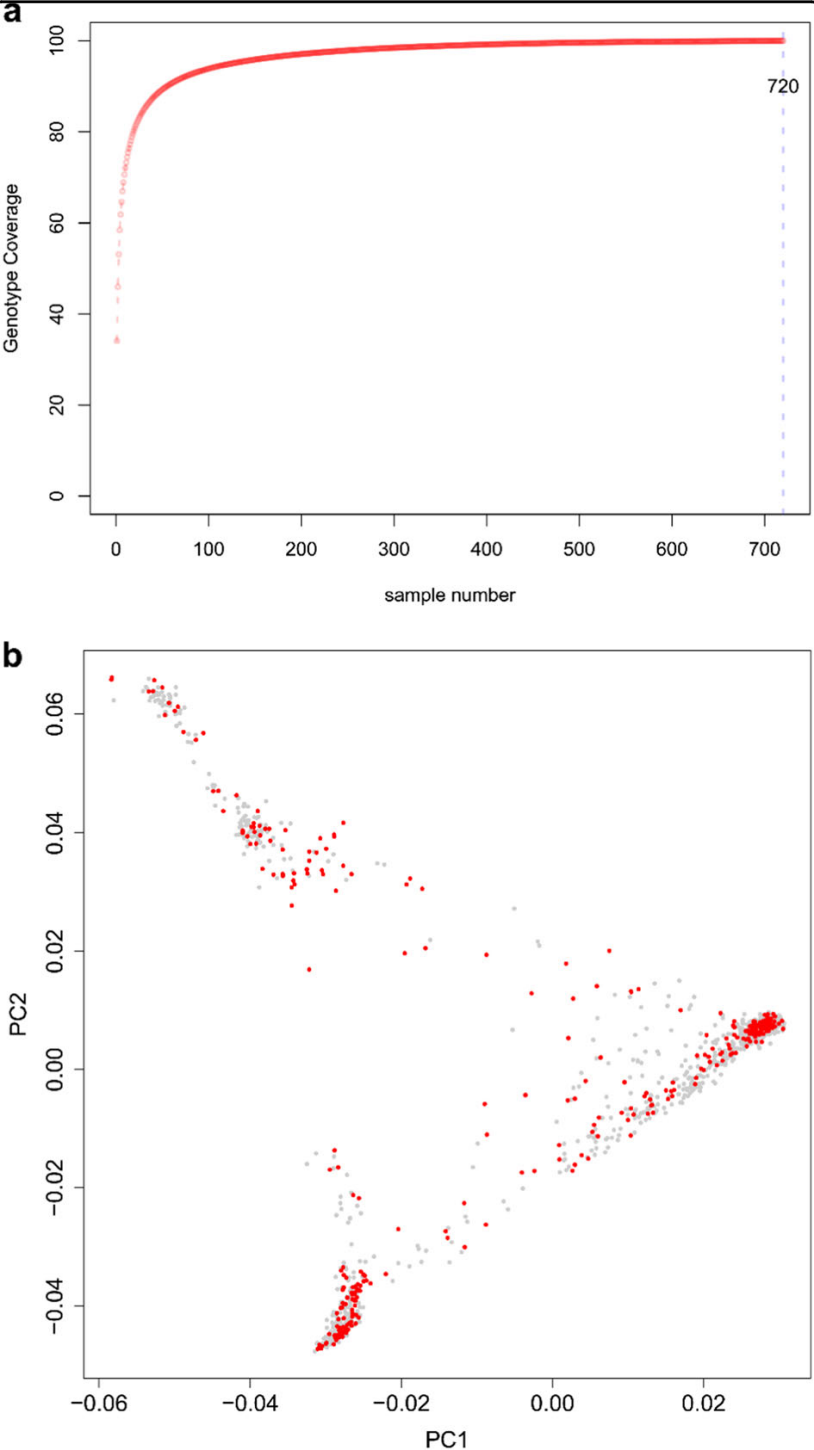
Development and evaluation of the cucumber core collection. **a** Coverage of allelic diversity versus number of selected accessions analyzed by GenoCore. **b** Principle component analysis (PCA) of cucumber accessions. Red dots: accessions in the core collection; gray dots: accessions not in the core collection

To develop this core collection, we first analyzed the 1234 cucumber accessions using the GenoCore program^42^. The results showed that the 720 top-ranked accessions captured 100% of the allelic diversity of the whole set, and the top 100, 200, 300, and 400 top-ranked accessions captured 93.87%, 97.09%, 98.47%, and 99.20% of the allelic diversity, respectively (Fig. 6a). According to this analysis, we first selected 354 accessions which captured 95.9% of the allele diversity in the collection of the 1234 cucumber accessions. Of the 354 accessions, 70 (19.8%) were from India/South Asia, 35 (9.9%) from Central/West Asia, 94 (26.6%) from East Asia, 10 (2.8%) from Africa, 20 (6.0%) from North America, 74 (20.9%) from Europe, 48 (13.6%) from Turkey, and 3 from other regions (Supplementary Table S6). PCA analysis of these 354 accessions in the core collection (Fig. 6b) showed the nearly identical patterns to those of the 1234 accessions in the entire collection.

An additional 41 historical varieties with important horticultural and disease resistance traits were added to this core collection, making the final core collection containing a total of 395 accessions. The additional accessions included some cucumber cultivars or germplasm that have played important roles in cucumber breeding in the US for the processing and fresh markets (Supplementary Table S6).

## Discussion

### Genetic characterization of the US NPGS cucumber collection

The genetic composition of the U.S. cucumber germplasm collection was characterized using high-throughput GBS analysis. A total of 1234 accessions, predominantly collected from India and South Asia, East Asia, Central and West Asia, Europe, North America, and Africa were genotyped, providing 279 K uniquely aligned sequence tags. From these data ~23.5 K biallelic SNPs representing minor alleles present in at least seven accessions (frequency > 0.01 and missing data rate < 0.5) were identified. With the exception of highly methylated centromeric or pericentromeric regions, the SNPs were well distributed at high density in the genome with an approximate frequency of 1 SNP per 10.6 kb. These data allowed for comprehensive analysis of phylogentic relationships, population structure, and LD patterns of accessions in the collection and provide a resource for genetic analysis and gene discovery.

Consistent with our current understanding about the geographic origin of cucumber^6^ and prior phylogenetic analyses^9,10^, the U.S. cucumber PI collection comprised three major clades. The basal clade predominantly comprised accessions from India/South Asia, the presumed center of domestication for *C. sativus*. While this clade had the deepest branches suggesting greater divergence among members of this clade, the overall diversity of accessions from this region was reduced relative to the study of Qi et al.^10^. This is likely due to sampling. The 216 accessions from India/South Asia in the U.S. collection included only three (1.4%) accessions of *C. s*. var. *hardwickii*, a highly diverse wild botanical variety believed to be either a progenitor or a feral relative of the cultivated cucumber, C. *s*. var. *sativus*^6,11,44^. In contrast, 13 of 30 accessions (43.3%) from the Indian accessions studied by Qi et al.^10^ were var. *hardwickii*.

From its origins in India and initial domestication ~3000 years ago, it appears that cucumber moved both East (to East Asia) and West (to Central and West Asia, Europe, Africa, and North America), following distinct trajectories in each case^27,45^. The strong differentiation between the East and West groups likely reflects a long period of divergent domestication (written Chinese records mentioning cucumber date as early as 164 BCE) as well as geographical isolation due to the Himalayan mountains^10,28,46^. Patterns of LD decay were consistent with the phylogenetic and population structure analyses. The Indian group had the shortest physical distance to reach half-maximal value (16 kb), vs. 32–48 kb for East Asia, Central/West Asia, and Europe. The reduced rate of LD for North American and Africa accessions (96 and 88 kb) may reflect the greater genetic relatedness of the samples in this collection, or the migration route for cucumber, which is thought to have been introduced into these regions comparatively recently from Europe^47^.

Several studies have indicated that the overall level of genetic diversity within cultivated cucumber is quite narrow, and that most of the genetic differentiation was observed between geographic regions or market classes^9^^,^^10^^,^^28^^,^^44^^,^^46^^,^^48^. Our phylogenetic analyses also reflected these sources of divergence. In addition to the separation observed among the three primary clades, we saw examples of differentiation within clades as evidenced by countries of origin, regions of collection within India, subgroups from Turkey, and between processing and fresh market cucumbers in North America. Among the Indian PIs, accessions from Madhya Pradesh, Uttar Pradesh, and Rajasthan were preferentially, but not exclusively, distributed in different subclades, suggesting diversity both within and between regions. Separation of accessions from Rajasthan relative to other regions in India was previously observed based on isozyme analysis performed following initial collection^44^. The current SNP-based analysis allowed for more nuanced assessment of relationships among the accessions. The Turkish germplasm also was associated with several subclades. For the two largest subclades containing Turkish accessions, one was extensively mixed with accessions from Central/West Asia, while the second was extensively mixed with accessions from Europe. Examination of collection locations within Turkey showed predominance of samples from the European-mixed subclade from western Turkey and samples from the Asian-mixed subclade from Eastern Turkey. There were some exceptions, however, possibly reflecting seed exchange across different regions of the country. Separation among the North American accessions reflected market class. As public and commercial breeding efforts have largely catered to either pickling or slicing cucumber, with delineated breeding efforts, it is not surprising to observe genetic divergence. Differentiation between pickling and slicing cucumbers also has been observed with RFLP markers and metabolomic analyses of cucumber fruit peels^28,49^.

### Development of genomic breeding tools

An important value of genetic characterization of the collection is the development of genomic tools for breeders. QTL analyses of key traits of economic importance can allow for the development of markers for marker assisted selection, focusing phenotypic selection on population subsets containing desired markers and facilitating gene pyramiding for complex traits. The GWAS presented here using the high-density SNP markers and historical phenotyping data for several disease resistance and physiological traits show the identification of significantly associated genomic regions. At this time QTL have been mapped for a limited number of traits in cucumber. Of the traits examined here, recent studies have reported QTL for downy mildew, GSB, and flowering time^50–56^.

Recent QTL mapping studies for downy mildew resistances in two PI lines (PI 330628 or WI 7120, and PI 197088) identified eight resistance QTL, *dm2.1, dm3.1, dm3.2, dm4.1, dm5.1, dm5.2, dm5.3*, and *dm6.1*. Among them, *dm2.1, dm4.1, dm5.2* and *dm6.1* seem to be shared by the two PI lines^50,51^. Another cucumber accession, PI 197087, possesses multiple resistances to downy mildew (pre-2004 strain, by the *dm1* locus), anthracnose (by the *cla* locus) and angular leaf spot (by the *psl* locus). Pan et al.^52^ and Wang et al.^54^ showed that *dm1/cla/psl* locus for the triple disease resistances in this PI line was controlled by the same *staygreen* gene (*CsSGR*), which was located in the short arm of chromosome 5 (~5 Mb in Gy14 V2.0). The several peaks on chromosome 5 detected from GWAS in this study (Supplementary Table S5) seem to correspond well to *dm1*, *dm5.1*, *dm5.2,* and *dm5.3* detected in Wang et al.^50,51,54^. In addition, the peak on chromosome 5 detected in GWAS for anthracnose resistance is likely the same as the *dm1/cla/psl* locus (CsSGR) originated from PI 197087 (ref.^52,54^). However, no downy mildew QTL was detected on chromosome 4 in the natural population, whereas no QTL on chromosome 7 were detected from bi-parental mapping populations, which was identified in GWAS. These differences may reflect the power of QTL detection with different approaches. The different virulence structure of field downy mildew pathogens may also contribute to the observed differences.

QTL for GSB resistance have been recently identified in cucumber and melon. Two QTL mapping studies on GSB resistances from the wild cucumber (*C.s*. var. *hardwickii*) accession PI 183967 have been reported with some contradictory results^53,55^. Liu et al.^55^ identified six QTL on chromosomes 3, 4, 5, and 6 (*gsb3.1, gsb3.2, gsb3.3, gsb4.1, gsb5.1, and gsb6.1*) with *gsb5.1* as a major QTL. On the other hand, Zhang et al.^53^ identified five QTL (*gsb-s1.1, gsb-s2.1, gsb-s6.1, gsb-s6.2*, and *gsb-s6.3*) for resistance to GSB in PI 183967 with *gsb-s6.2* having the largest effect. While not directly overlapping, *gsb-s2.1* (ref.^53^) and *gsb5.1* (ref. ^55^) seem to be at nearby regions of the two peaks we identified from GWAS in this study on chromosomes 2 and 5, respectively, which obviously need further investigation to confirm. In addition, a recent report also identified a candidate gene for GSB resistance in melon located on chromosome 4 around 4.0 Mb^57^; however, it does not appear to reside in syntenic regions^58^ with the QTL identified this study.

QTL for flowering time was previously mapped on chromosomes 1, 2, 5, and 6 in recombinant inbred lines derived from a cross between an American pickling type and little leaf (ll) line H-19 (ref. ^56^), and on chromosomes 1, 5, and 6 in a cross between American pickling cucumber and semi-wild var. *Xishuangbanna*^16^. There appears to be potential overlap among the identified QTL in those studies and the current GWAS. In all three studies significant regions were located on the distal end of chromosome 1 and on the central region of chromosome 6, suggesting potentially robust loci influencing flowering time over a range of genetic backgrounds.

Genetic characterization of accessions within a germplasm collection and knowledge of their genetic relationships also enables definition of a core population, i.e., a subset of the full collection that captures the majority of diversity of the species^29,59^. Core collections can greatly facilitate breeding and preservation efforts by providing a common starting point for screening the population for traits of importance for crop improvement. By allowing for reduced numbers in the initial screening stages, they can be especially helpful when phenotyping a trait of interest that is particularly expensive or labor-intensive. A defined core population also can allow for more focused management of seed supplies for distribution. While core populations can be defined using geographic or phenotypic characteristics, establishment of maximally valuable core populations, relies on effective measures of genetic diversity among the accessions^29^.

A prior core of 147 accessions from the NPGS cucumber collection was proposed based on isozyme analysis of 970 PIs, along with data regarding disease resistance (angular leaf spot, anthracnose, downy mildew, rhizoctonia fruit rot, and target leaf spot), water and heat stress tolerance, and morphological characteristics^45^. Current next-generation sequencing technology allows for more robust genotypic assessment. From the analyses performed here, we have designed a core collection of 354 accessions that represent 96% of the genetic variation present in the NPGS. Approximately half (76) of the PIs from the prior core^45^ were included in the current core collection. It has also been recommended that germplasm collections include important breeding materials where key traits have been introgressed into cultivated inbred lines^4,29^. To this end, the proposed core also includes 41 accessions, including historical cultivars, widely used breeding lines and individuals with identified traits of interest. To make the core maximally valuable for future breeding efforts and genetic studies, we are in the process of deep resequencing of the genomes, and creating seed stocks of the selected accessions in the final core collection, under the current USDA CucCAP project (https://cuccap.org/). Both the genotype data and seeds of the core collection will be accessible to the public.

## Conclusions

This work has provided detailed genetic analysis of the cucumber germplasm collection maintained by the US NPGS, which includes more than 1200 accessions collected throughout the world. The information provided by the GBS data has provided deep insight into the diversity present within the collection and genetic relationships among the accessions. These data can be used for genetic analyses such as GWAS to identify potential genomic regions associated with valuable traits, and for informed management of the collection to conserve genetic resources. Development of the genetically informed core collection will enable more efficient genetic analyses that can be coupled with sophisticated genomic tools to facilitate crop improvement. While it is clear that a great deal of valuable diversity is represented among the materials in the NPGS collection, these observations also illustrate the importance of careful and extensive germplasm collection to ensure that our collections reflect the extant diversity available worldwide.

## Electronic supplementary material

Supplementary Note and Figures

Supplementary File 1

Supplementary Tables

## Data Availability

Raw GBS reads for all individual cucumber accessions have been deposited in the NCBI sequence read archive (SRA) under accession numbers SRP149275 and SRP149431. Raw and filtered SNPs in VCF format are available at ftp://cucurbitgenomics.org/pub/cucurbit/GBS_SNP/cucumber.
