# Supplementary material for: The USDA cucumber (Cucumis sativus L.) collection: genetic diversity, population structure, genome-wide association studies, and core collection development
Source: Hortic Res. 2018 Oct 1;5:64. doi: 10.1038/s41438-018-0080-8 (PMC6165849; doi:10.1038/s41438-018-0080-8)
Supplement: Supplementary file 1 — Supplementary Note and Figures [file 41438_2018_80_MOESM1_ESM.pdf]

Supplementary note

Phenotypic data collection

Over the last 30 years, cucumber (*C. sativus* L.) data for yield, fruit quality, and resistance to biotic and abiotic stresses have been collected at the Cucurbit Breeding program of North Carolina State University. For GWAS in this study, we used historical data of 13 agronomic traits of cucumber plant introduction (PI) collected over multiple years and locations (http://cucurbitbreeding.com).

Data for resistance to anthracnose, downy mildew and gummy stem blight was collected 2 to 4 weeks after inoculation. Plants were weekly rated at least 6 times for foliar lessons using a visual scale of 1 to 9, where 0 = none, 1-2 = trace, 3-4 = slight, 5-6 = moderate, 7-8 = severe, and 9 = plant dead[1-3].

Data for root knot nematode resistance (*Meloidogyne hapla* race 1, *M. arenaria* race 2, or *M. incognita* race 3) was collected on washed roots 9 weeks after inoculation (11 weeks after planting) using the gall index system, where 0% = immune, 1% to 10% = highly resistant, 11% to 20% = moderately resistant, 21% to 40% = slightly resistant, and >40% = susceptible[4-5].

Data for fruit shelf life (fruit weight loss, firmness loss and shriveling). Fruit weights were measured before and 2 weeks after storage; and measure in percent loss. Shivering was also rated 2 weeks after storage using a visual scale of 0-9 with 0= none (no appearance of shriveling of fruit skin), 1-3=slight, 4-6=moderate, and 7-9= severe (skin very shriveled). Firmness was tested as amount of force (Newton) required to penetrate the exocarp and mesocarp of fruit. Firmness was tested before and 2 weeks after storage; and measure in percent loss[6].

Data for yield was collected as plot means and consisted of number and weight of total fruits per plot. Yield was expressed as thousands of fruits ha^{-1} for fruit number, and Mg ha^{-1} for fruit weight to make comparisons with other studies easier[7].

Flowering data was taken for 21 days after the appearance of the first staminate flower on the earliest plant. Number of days from planting to first staminate flower was recorded for each plant. Pollinations were made on the earliest and latest cultigens to observe fruits, skin and spine type[8].

Root size data was collected 3 months after planting. Roots were washed free of soil using tap water and all plant roots were measured. Root lengths were obtained by stretching roots to the

same width (150 mm) and height (5 mm), and then measuring the length of the root system in mm[9].

Data for chilling tolerance was collected after plants at 2nd to 3rd true leaf stage were subjected to a chilling treatment of 3, 5, 7, and 9 hours at 4 °C a light intensity of approximately 500 µmol × M^{-2} × s^{-1} PPFD 400 to 700 nm. Leaf damage was rated on a scale of 0 to 9, where 0 = no damage, 1–2 =trace, 3–4 = slight, 5–6 = moderate, 7–8 = advanced, 9 = dead.

All data was summarized as the average of several ratings or data points.

References

1. Wehner, T.C. & St. Amand, P.C. Anthracnose resistance of the cucumber germplasm collection in North Carolina field tests. *Crop Sci.* **35,** 228–236 (1995).
2. Wehner, T.C. & Shetty, N.V. Downy mildew resistance of the cucumber germplasm collection in North Carolina field tests. *Crop Sci.* **37,** 1331–1340 (1997).
3. Wehner, T.C. & Shetty, N.V. Screening the cucumber germplasm collection for resistance to gummy stem blight in North Carolina field tests. *HortScience* **35,** 1132–1140 (2000).
4. Wehner, T.C., Walters, S.A. & Barker, K.R. Resistance to root-knot nematodes in cucumber and horned cucumber. *J. Nematol.* **23,** 611–614 (1991).
5. Walters, S.A., Wehner, T.C. & Barker, K.R. Root-knot nematode resistance in cucumber and horned cucumber. *HortScience* **28,** 151–154 (1993).
6. Wehner, T.C., Shetty, N.V. & Wilson, L.G. Screening the cucumber germplasm collection for fruit storage ability. *HortScience* **35,** 699–707 (2000).
7. Shetty, N.V. & Wehner, T.C. Screening the cucumber germplasm collection for fruit yield and quality. *Crop Sci.* **42,** 2174–2183 (2002).
8. Walters, S.A. & Wehner, T.C. Evaluation of the United States cucumber germplasm collection for early flowering. *J. Plant Breeding* **112,** 234–238 (1994).
9. Walters, S.A. & Wehner, T.C. Evaluation of the U.S. cucumber germplasm collection for root size using a subjective rating technique. *Euphytica* **79,** 39–43 (1994).

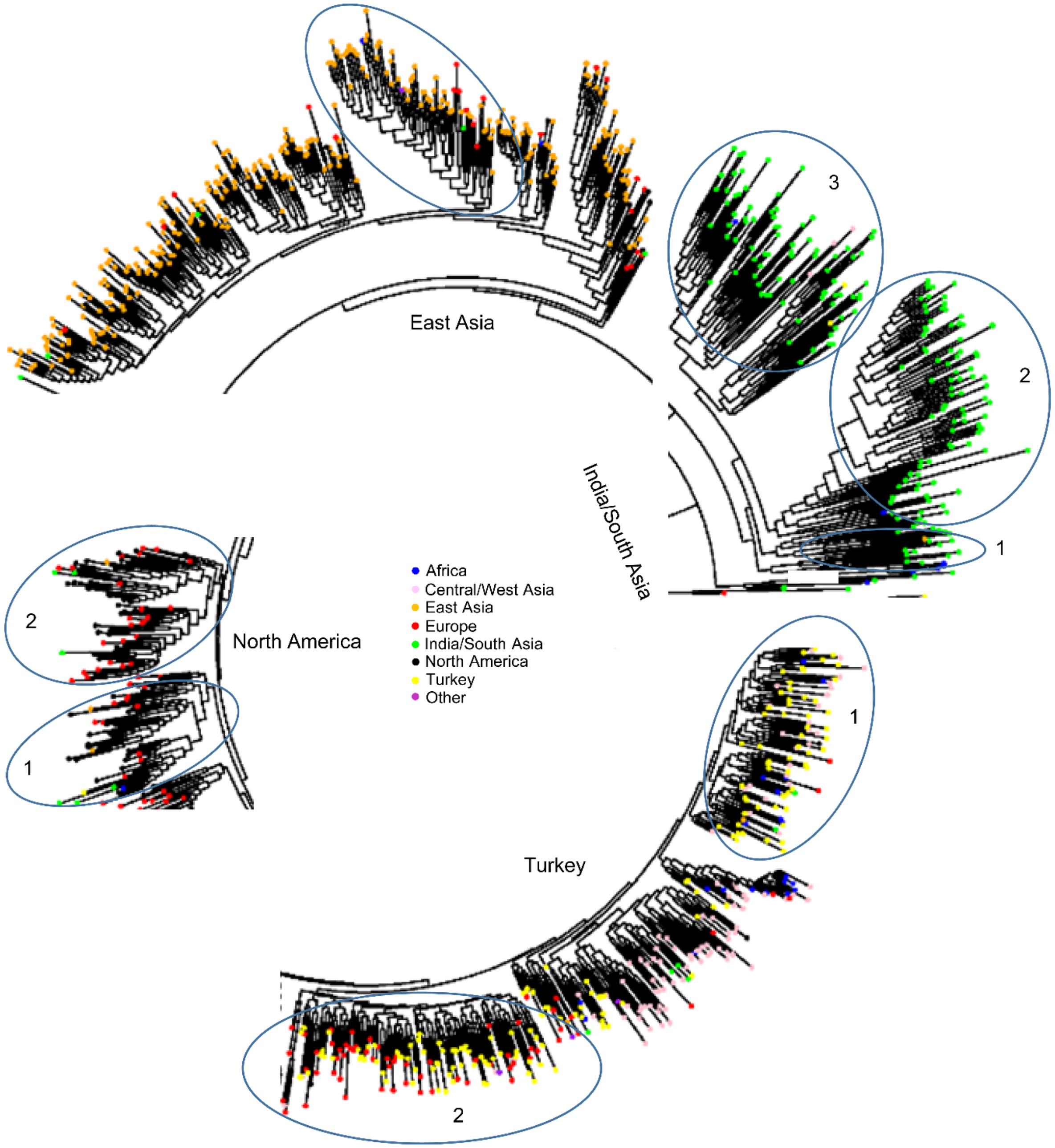

Supplementary Fig. S1. Subclades of cucumber accessions from different regions of the world. In the East Asia group, accessions from Japan and Korea were primarily included in the circled sub-clade. For accessions from Turkey, those in Subclade 1 were mixed with accessions from Central/West Asia and those in subclade 2 were mixed with accessions from Europe. For accession from North America, those in subclade 1 predominantly included slicing cucumbers, and those in subclade 2, pickling cucumbers.

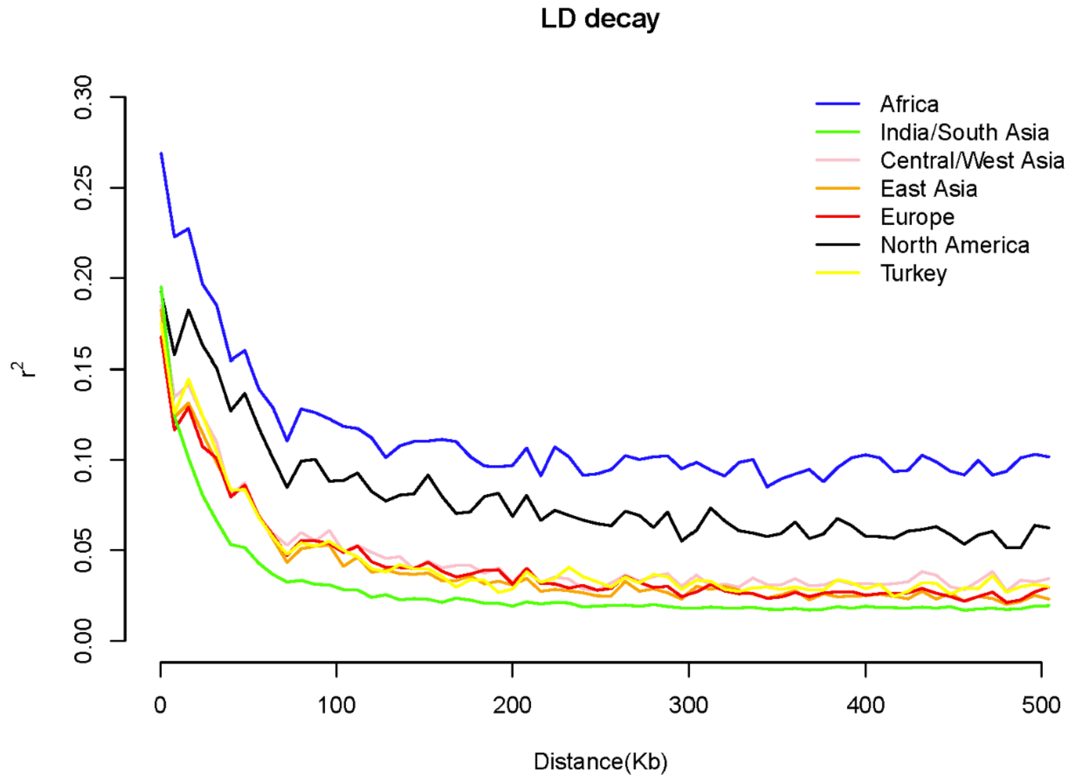

Supplementary Fig. S2. Linkage disequilibrium (LD) of cucumber accessions. LD decay was determined by squared correlation of allele frequencies (r^2) between pairwise SNPs in each group**.**

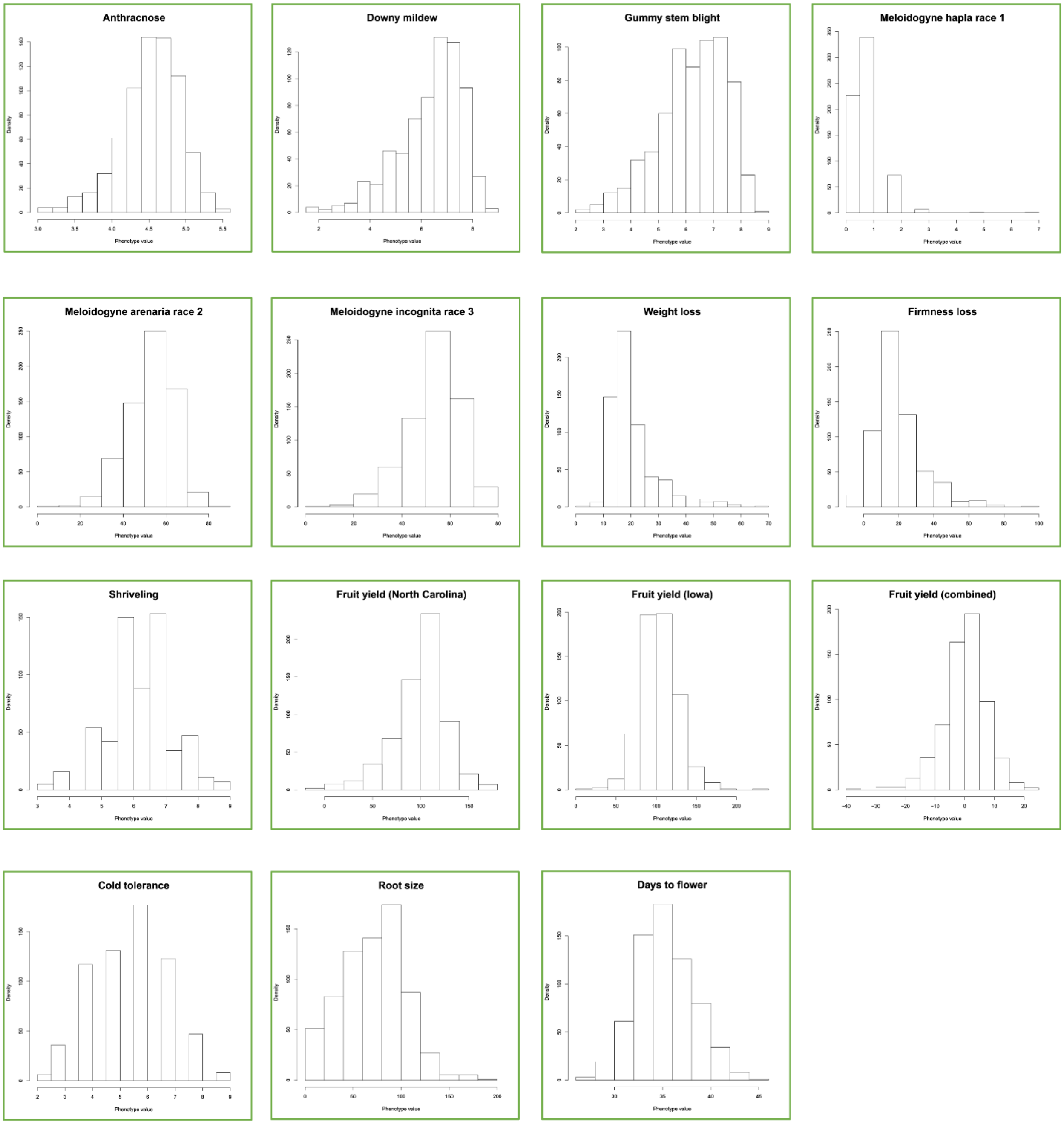

Supplementary Fig. S3. Frequency distribution of different traits in cucumber accessions genotyped in the current study.

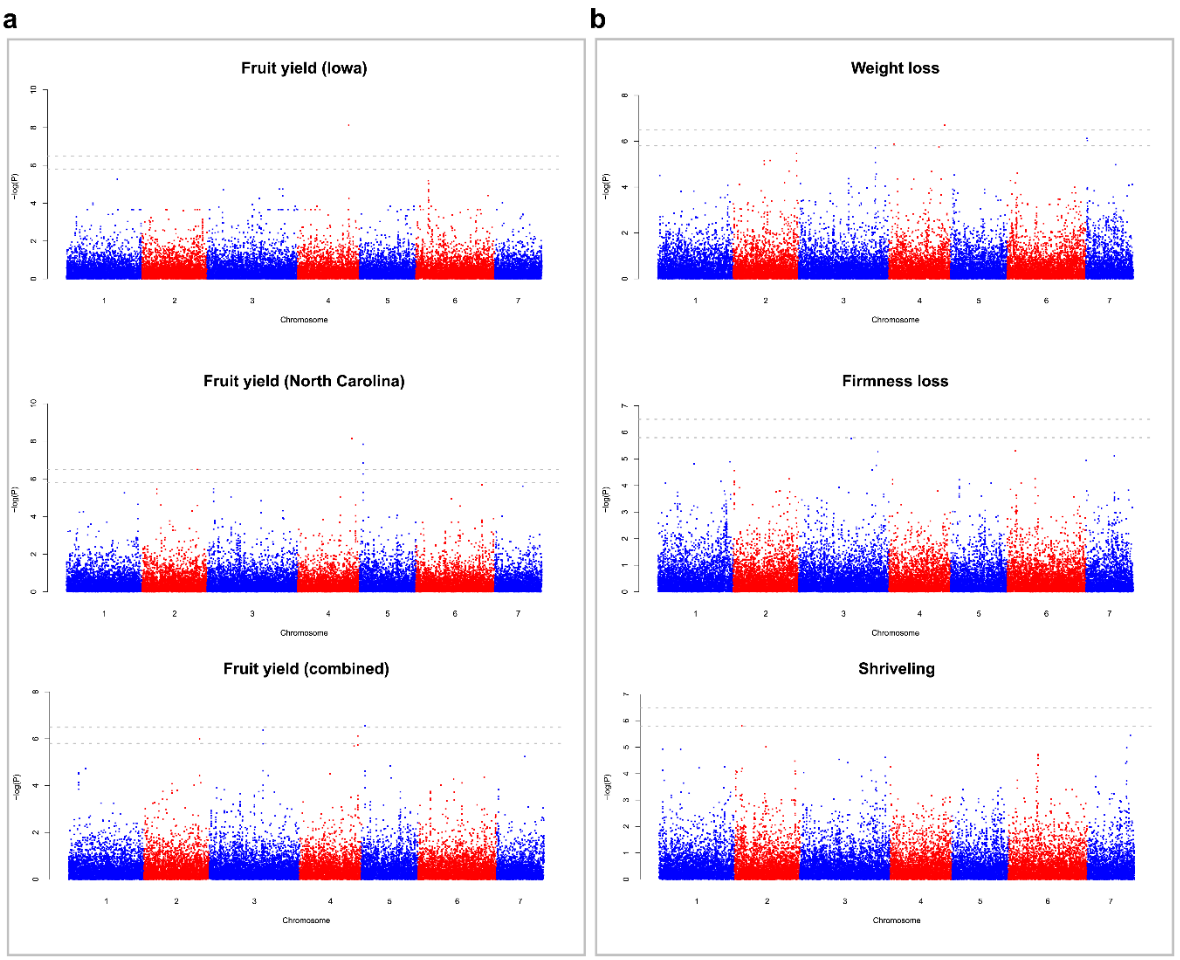

Supplementary Fig. S4. Manhattan plot of genome-wide association study (GWAS) results. **a,** GWAS for fruit yield trait. **b,** GWAS for fruit shelf life traits including % weight loss, % firmness loss, and shriveling.

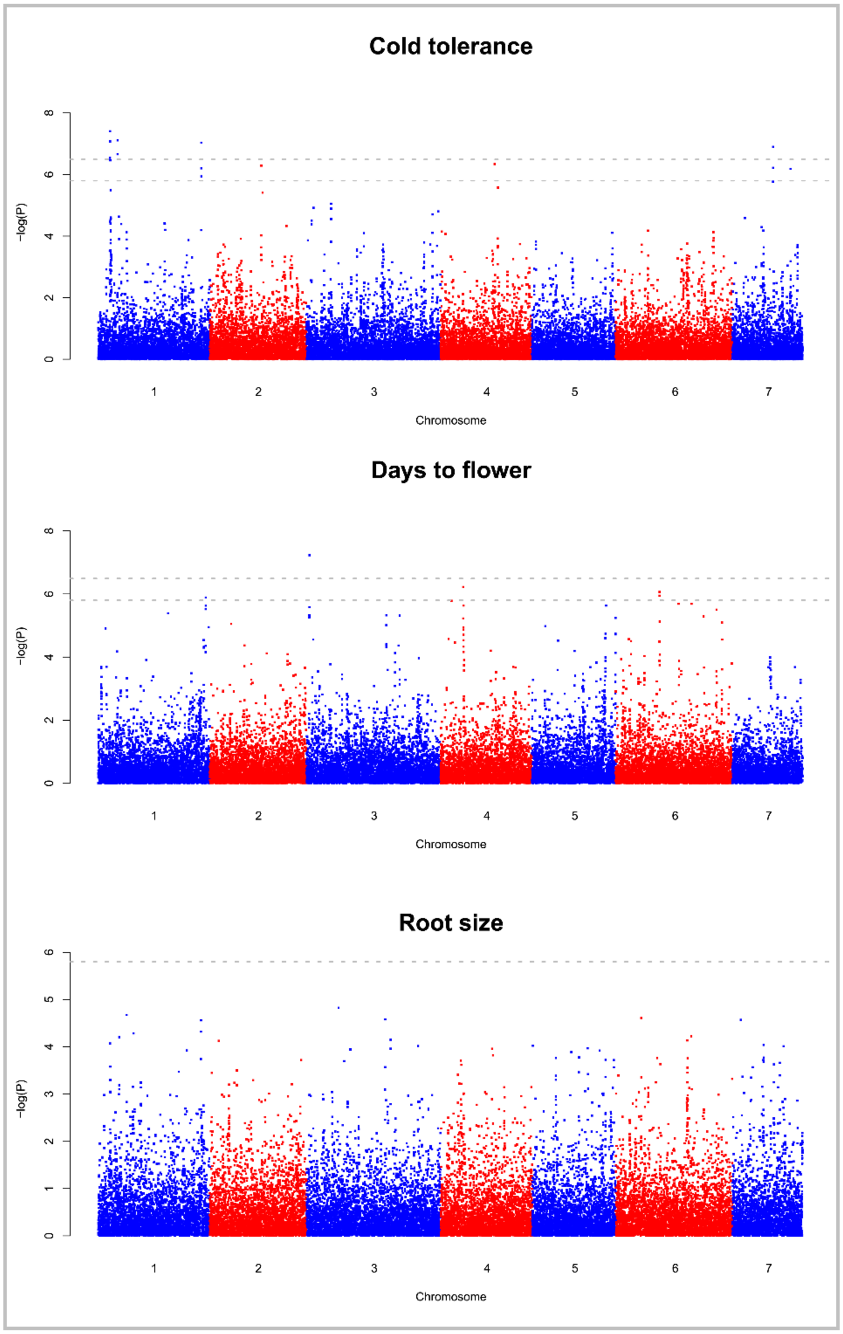

Supplementary Fig. S5. Manhattan plot of genome-wide association study (GWAS) results for chilling tolerance, days to flower, and root size traits.
