# Supplementary figures and images for: The USDA cucumber (Cucumis sativus L.) collection: genetic diversity, population structure, genome-wide association studies, and core collection development
Source: Hortic Res. 2018 Oct 1;5:64. doi: 10.1038/s41438-018-0080-8 (PMC6165849; doi:10.1038/s41438-018-0080-8)

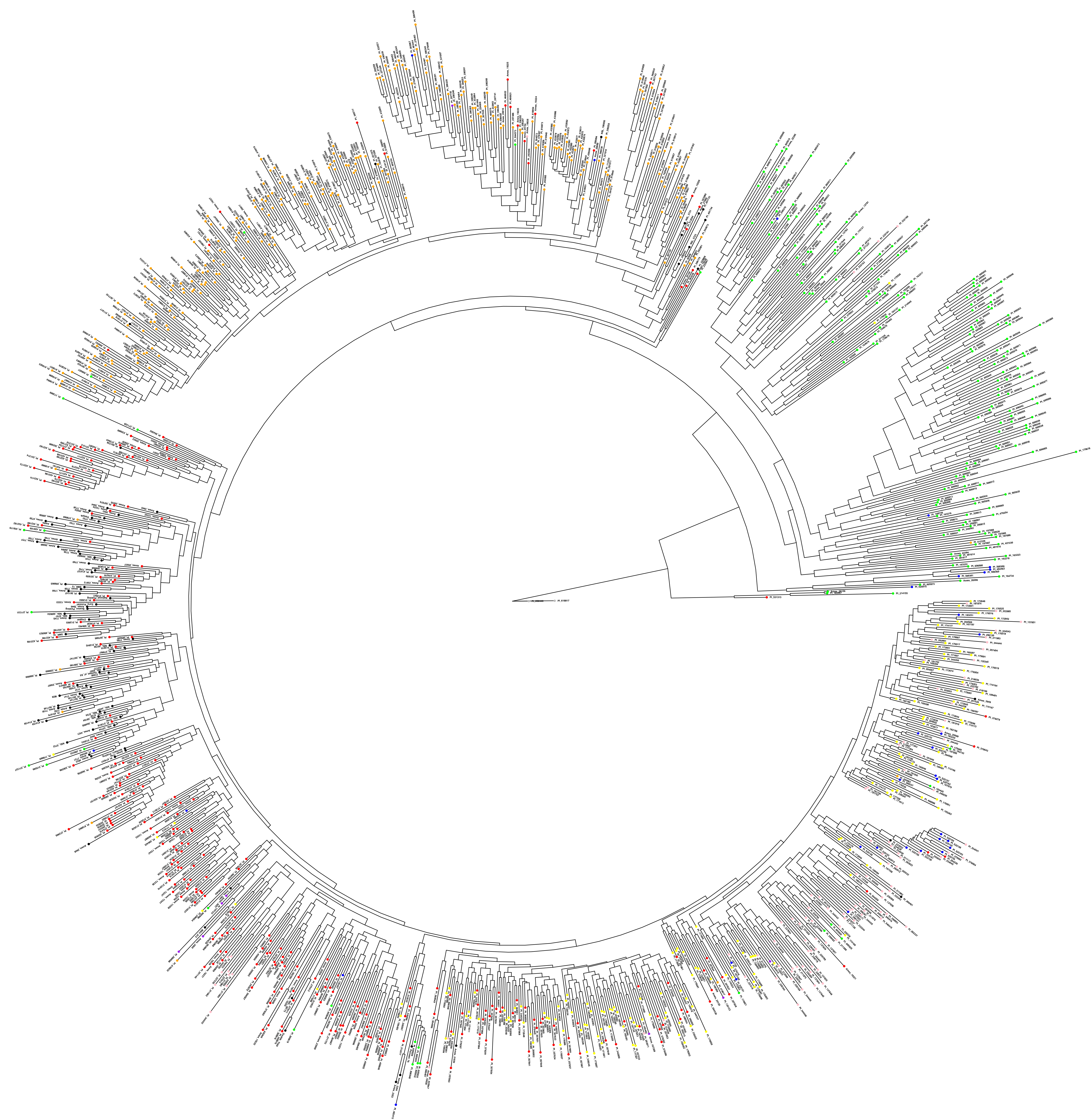

Supplement: Supplementary file 2 — Supplementary File 1 [file 41438_2018_80_MOESM2_ESM.pdf]
